# Combination of cadonilimab (PD-1/CTLA-4 bispecific antibody) and apatinib as salvage therapy achieves partial response in MSI-H advanced gastric cancer: a case report

**DOI:** 10.3389/fimmu.2025.1533700

**Published:** 2025-02-18

**Authors:** Jiayu Zhao, Xiaoxiao Li, Xiaoyuan Sun, Ruoxi Xiao, Juan Xue, Kexin Sui, Zimin Liu

**Affiliations:** ^1^ Department of Oncology, Shouguang People’s Hospital, Weifang, China; ^2^ Digestive Tumor Treatment Center, Affiliated Hospital of Qingdao University, Qingdao, China; ^3^ Department of Medcine, Qingdao University, Qingdao, China; ^4^ Department of Medcine, Shandong First Medical University, Affiliated Hospital of Qingdao University, Jinan, China

**Keywords:** PD-1/CTLA-4 bispecific antibody, AK104/cadonilimab, microsatellite instability, apatinib, salvage therapy, advanced gastric cancer

## Abstract

Microsatellite instability-high (MSI-H) gastric cancer (GC) is recognized as a unique subtype of gastric cancer. While patients with advanced MSI-H gastric cancer may respond favorably to a combination of immune checkpoint inhibitors and chemotherapy in first-line treatment, no definitive recommendations exist regarding the optimal regimen for subsequent therapy. Cadonilimab, a PD-1 and CTLA-4 bispecific antibody, has shown encouraging efficacy and safety in the first-line treatment of advanced gastric cancer. However, its utility in the MSI-H gastric cancer subtype following multiple lines of therapy remains uncertain. This case report describes a patient with advanced MSI-H gastric adenocarcinoma that progressed after multiple treatments and achieved notable efficacy with a combination of cadonilimab and apatinib. By examining the current therapeutic landscape for MSI-H gastric cancer, this study explores the potential of combining PD-1/CTLA-4 dual-immunity with anti-vascular therapy as salvage treatment for this gastric cancer subtype. The findings provide valuable reference points for future clinical trials, offering a promising perspective on backline therapeutic strategies for MSI-H gastric cancer and highlighting the potential of integrating bispecific antibodies with anti-vascular therapies.

## 1 Introduction

GC is a prevalent malignant tumor of the digestive system, with MSI-H gastric cancer recognized as a distinct subtype characterized by significant sensitivity to immunotherapy. Based on the KEYNOTE-062 study, the recommended first-line treatment for advanced MSI-H gastric cancer includes either single-agent immunotherapy or immuno-combination chemotherapy (1). For advanced patients who have not previously received immunotherapy, PD-1/PD-L1 monoclonal antibodies remain a standard option for second-line treatment (2). However, if an immune checkpoint inhibitor was used as first-line therapy, subsequent treatment is selected based on HER2 status, involving anti-angiogenic drugs combined with antibody-drug conjugates (ADCs) or additional immune checkpoint inhibitors. The rarity of MSI-H gastric cancer limits the availability of high-quality, large-scale evidence to guide backline treatment strategies. Particularly in HER2-negative MSI-H gastric cancer, there is a lack of established recommendations for therapy following resistance to PD-1/PD-L1 inhibitors.

Cadonilimab, a bispecific antibody targeting both PD-1 and CTLA-4, has demonstrated strong efficacy in the first-line treatment of advanced gastric cancer, as reported in the GEJCIb clinical trial (3). Ongoing clinical trials are investigating its role in the second-line treatment of MSI-H tumors(NCT04556253). Apatinib, an anti-angiogenic agent, is approved in China for third-line and beyond treatment of advanced gastric adenocarcinoma. It functions by inhibiting tumor angiogenesis, increasing T-cell infiltration in tumors, and improving the suppressive tumor microenvironment, thereby facilitating increased T-cell penetration into tumor tissue (4–6). When combined with PD-1/CTLA-4 bispecific antibodies, apatinib can amplify the anti-tumor activity of infiltrating T cells and boost tumor cell sensitivity to immunotherapy. Although promising, the efficacy of cadonilimab combined with anti-angiogenic agents in treating advanced MSI-H tumors requires validation in larger clinical trials. This study presents a case of a patient with advanced MSI-H gastric cancer resistant to PD-1 monoclonal antibody therapy who achieved partial remission(PR) and PFS exceeding 20 months following treatment with cadonilimab and apatinib. The purpose of this study is to evaluate the current therapeutic landscape for MSI-H gastric cancer and explore the potential utility of cadonilimab combined with apatinib in salvage treatment for this subtype.

## 2 Case description

**Figure 1:**
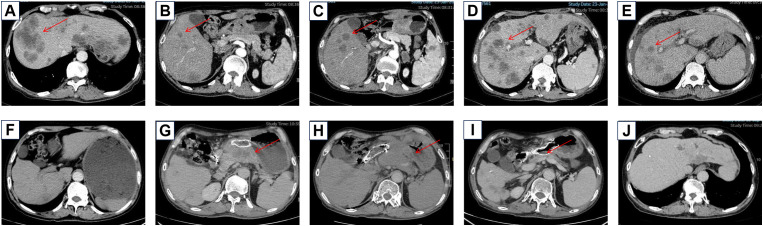
Baseline imaging of liver metastases before treatment **(A)** Baseline imaging of liver metastases before treatment **(B)** Liver metastases increased after SOX chemotherapy **(C)** Liver metastases before ICI treatment **(D)** Liver metastases decreased after ICI treatment **(E)** Gastric retention was found during the maintenance treatment of Herceptin and ICI **(F)** The primary focus progressed after the maintenance treatment of Herceptin and ICI **(G)** The primary focus continued to progress after the treatment of vidiximab combined with ICI **(H)** The primary focus improved after the treatment of cardenimab combined with apatinib **(I)** The liver metastasis was reduced after the treatment of cardenimab combined with apatinib **(J)**.

A 59-year-old male with a history of smoking and alcohol consumption presented with non-specific upper abdominal pain lasting five months, accompanied by melena for one month. Gastroscopy revealed a lesion in the gastric sinus, and histopathology confirmed a moderately differentiated carcinoma. The patient had an ECOG performance score of 0 and was in good overall condition. Enhanced CT scans identified metastases in the abdominal and retroperitoneal lymph nodes, both lungs, liver, and the left adrenal gland (**Figures 1A, B**). Due to the lack of surgical indications, the patient was initiated on two cycles of SOX regimen chemotherapy(L-OHP 130mg/m2 d1+S-1 40mg/m2 bid d1-14,q3w). Pathological samples were sent for NGS, which identified mutations in ERBB2,KRAS2,PIK3CA,POLE,POLD1and BRCA2; a tumor mutational burden (TMB) of 62.1; microsatellite instability-high (MSI-H); and PD-L1 expression levels with a TPS of 10% and a CPS of 11. In light of the ERBB2 mutation, Herceptin(Loading dose 8mg/kg, Maintenance dose 6mg/kg,q3w) was added to the SOX regimen starting from the second cycle of chemotherapy.

The patient was reviewed after every two cycles of chemotherapy, with efficacy evaluated according to RECIST criteria and adverse events assessed using CTCAE 5.0 criteria. Following two cycles, the patient experienced no significant side effects. However, imaging showed enlargement of the left adrenal metastasis, indicating disease progression (**Figure 1C**). The treatment was subsequently adjusted to a regimen of four cycles of albumin-bound paclitaxel combined with capecitabine (albumin-bound paclitaxel 125mg d1,d8+capecitabine 1000mg/m2 bid d1-14,q3w), while Herceptin therapy was continued. Considering the patient’s MSI-H status, nivolumab immunotherapy(360mg,q3w) was introduced (**Figure 1D**). During this phase, the adrenal metastases showed significant reduction, while liver metastases exhibited slight regression, and the overall response was assessed as PR. The patient reported improvement in epigastric pain but experienced decreased appetite, hair loss, and grade II leukopenia. Maintenance therapy with Herceptin and nivolumab was continued, and follow-up evaluations were conducted every six months (**Figure 1E**). After seven months of maintenance therapy, the patient reported significant abdominal distension and poor dietary intake. Enhanced abdominal CT revealed mild progression of the primary gastric lesion with gastric retention (**Figure 1F**), while the remaining metastatic sites showed no notable changes. Comprehensive evaluation determined the disease status to be stable. To address the gastric retention, gastric sinus stenting was performed by the Department of Interventional Medicine, and Herceptin combined with nivolumab therapy was continued. Following stent placement, the patient experienced significant relief from abdominal distension and improved appetite.

**Figure 2:**
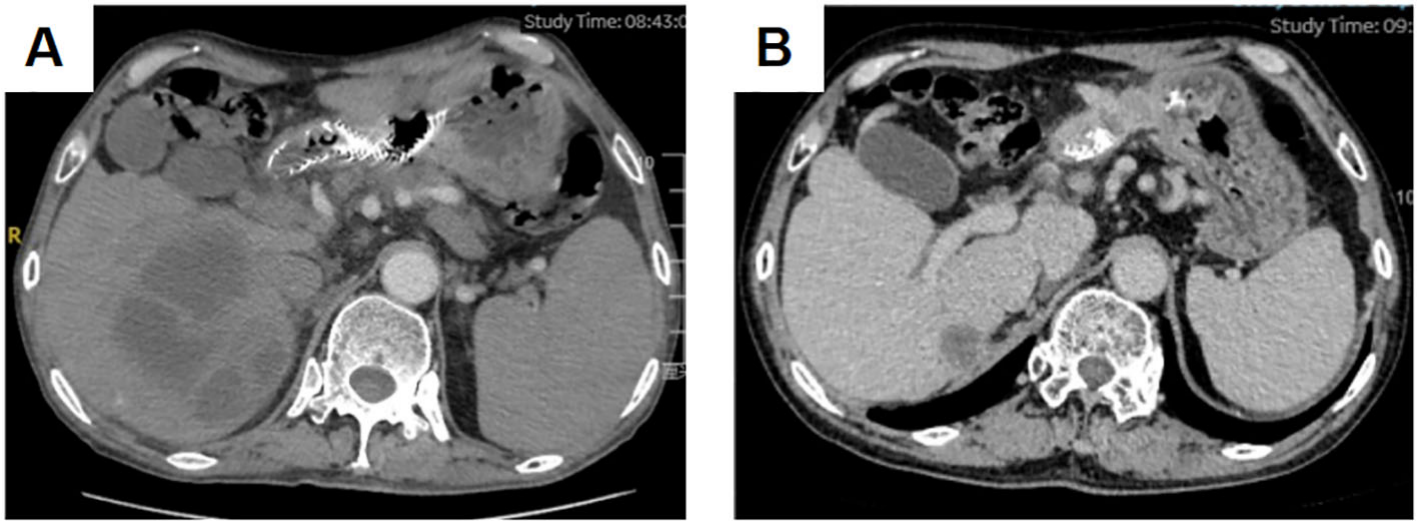
Liver abscess before drainage **(A)** Liver abscess after drainage **(B)**.

**Figure 3:**
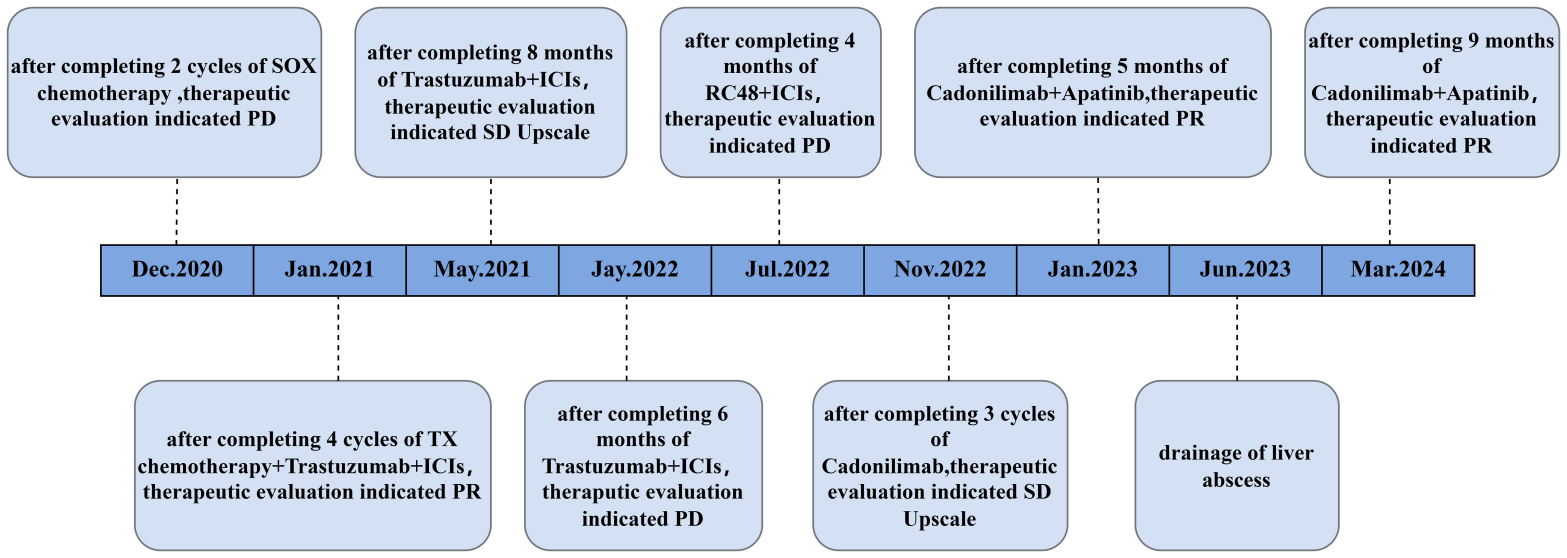
Timelines of events.

The gastric sinus primary tumor, retroperitoneal lymph nodes, and liver metastases showed progression upon review after 5 months (**Figure 1G**). Given the resistance to Herceptin, third-line treatment was initiated with the anti-HER2 ADC drug vedotin (2.5mg/kg,q2w) in combination with nivolumab(3mg/kg,q2w). During this period, the patient experienced poor appetite and persistent vague epigastric pain. A follow-up examination 3 months later revealed continued progression of the primary lesion in the gastric sinus (**Figure 1H**). As the disease advanced further, nivolumab resistance was suspected, prompting a switch to fourth-line therapy with the anti-PD-1/CTLA-4 bispecific antibody cadonilimab(6mg/kg,q3w). After 3 months of cadonilimab monotherapy, slight enlargement of the primary lesion, hepatic metastases, and lymph nodes was observed. The overall evaluation was SD (enlarged). Consequently, apatinib(850mg,qd) was added to the treatment regimen. Six months of combined cadonilimab and apatinib therapy showed notable improvement in the primary lesion and hepatic metastases, with the response assessed as PR (**Figures 1I, J**). However, CT scans revealed a new cystic hypodense lesion in the right liver lobe measuring 108 mm × 96 mm (**Figure 2A**). The enhancement scan showed mild strengthening of the cystic wall and septum. Concurrently, the patient developed a fever of 38.7°C and experienced right upper abdominal discomfort. Based on CT findings, a liver abscess was diagnosed. Ultrasound-guided transhepatic puncture and drainage of the liver abscess were performed, along with antibiotic therapy. Approximately 300 mL of purulent fluid was drained, and bacterial culture identified Klebsiella pneumoniae. The patient recovered well following the intervention (**Figure 2B**). Twenty months after initiating the combination therapy, the patient reported significant improvement in symptoms. CT scans showed no significant changes in the primary lesion compared to previous imaging, while liver metastases were significantly reduced, and retroperitoneal lymph nodes and adrenal metastases had completely resolved. The patient’s condition will continue to be monitored. The changes of tumor markers and lesions in patients are shown in **Table 1**. The timeline of the patient is summarized in **Figure 3**.

**Table 1:** Chronological evolution of clinical and biological parameters.

	Time node					
	2020-11(initial treatment)	2021-01(progress after SOX)	2021-05(TX+ICI+ Herceptin)	2022-07(progress after ICI+ Herceptin)	2022-11(progress after vidiximab + ICI)	2024-06(Cadonilimab+ apatinib)
Tumor markers						
CEA (ng/L)	60.92	183.20	3.72	6.25	7.26	5.02
CA-724 (U/ml)	406.00	617.00	9.02	60.50	88.84	7.67
CA-242 (U/ml)	7114.00	9605.00	41.28	72.75	113.30	26
CA-199 (U/ml)	4772.00	15425.00	85.20	122.62	161.30	136.60
Focus						
The long diameter of liver metastases (mm)	102	105	70	43	38	22
Primary focus of gastric wall	Thickened	No obvious change compared with 2020-11.	Better than 2021-01	Obviously thicker than 2021-05	Thicker than 2022-07	Obviously improved compared with 2022-11

## 3 Discussion

Gastric cancer is a prevalent malignant tumor of the digestive system and ranks fourth in cancer-related mortality. It exhibits significant heterogeneity and can be categorized into four subtypes based on cancer genome mapping: EBV-positive tumors, microsatellite unstable (MSI) tumors, genome-stable tumors, and chromosomally unstable tumors. Among these, the MSI subtype is notably sensitive to immunotherapy. Microsatellites (MS) are short, tandemly repeated DNA sequences that play a critical role in gene replication and expression (7). The mismatch repair (MMR) system ensures the stability of MS and facilitates accurate DNA replication. When defects occur in the MMR system (dMMR), they lead to the MSI-H phenotype, characterized by genomic instability and a high tumor mutational burden. Furthermore, MS regions are rich in frameshift mutations that can produce a significant number of neoantigens, which are closely linked to increased immune cell infiltration (8, 9). Due to the high mutational burden and the unique immune microenvironment of MSI-H gastric cancer, ICIs are capable of eliciting effective and durable immune responses against these tumors. In this case, the patient was diagnosed with gastric adenocarcinoma through gastroscopy and pathological examination, and NGS confirmed the presence of microsatellite instability. This established the diagnosis of advanced gastric adenocarcinoma of the MSI-H subtype.

### 3.1 Chemotherapy for MSI-H gastric cancer

In this case, the patient’s disease progressed rapidly after receiving two cycles of first-line chemotherapy with an oxaliplatin-capecitabine regimen. Current research indicates that MSI-H patients often exhibit elevated thymidylate synthase expression, which can lead to resistance to 5-fluorouracil (5-FU)-based therapies (10). This resistance may explain why fluorouracil monotherapy offers limited benefit to patients with MSI-H gastric cancer. Furthermore, the tumor microenvironment of MSI-H gastric cancer is immunosuppressive, and chemotherapy may exacerbate this suppression, potentially contributing to the rapid disease progression observed in this patient during first-line chemotherapy.

Despite these challenges, chemotherapy remains a critical component in the management of progressive gastric cancer. For MSI-H/dMMR gastric cancer, the comparative efficacy of single-agent ICIs versus ICIs combined with chemotherapy remains uncertain. Chemotherapy has been shown to induce immunogenic cell death in tumor cells, which activates dendritic cells and CD8+ T cells (11). Therefore, combining ICIs with chemotherapy may help overcome the initial resistance of MSI-H gastric cancer to immunotherapy. In the KEYNOTE-062 trial, the subgroup analysis for MSI-H tumors revealed that the ORR and PFS were higher in patients treated with pembrolizumab combined with chemotherapy compared to those receiving pembrolizumab monotherapy. However, the pembrolizumab monotherapy group displayed a more favorable trend in overall survival, while the combination therapy group demonstrated slightly less favorable survival outcomes. Although the trial did not directly compare the efficacy of ICIs alone versus ICIs combined with chemotherapy, the findings suggest that chemotherapy in combination with ICIs may provide an initial therapeutic advantage for MSI-H/dMMR patients. The long-term benefit of combining chemotherapy with ICIs remains unclear and warrants further investigation in future clinical trials.

### 3.2 Gene mutations and molecularly targeted therapies

The patient was diagnosed with HER2-positive advanced gastric cancer following NGS results that identified a mutation in the ERBB2 gene. Based on this diagnosis, the patient underwent anti-HER2 targeted therapy with trastuzumab during both first- and second-line treatments. Data from the C008 study demonstrated that vedotin achieved an ORR of 24.8%, a mPFS of 4.1 months, and a mOS of 7.9 months (12). Furthermore, vedotin has shown efficacy in patients with low HER2 expression. Based on these findings, the anti-HER2 ADC vedotin was administered as third-line therapy. However, the patient’s disease progressed rapidly after this treatment. The diagnostic criteria for HER2 positivity typically involve immunohistochemical testing, showing HER2 protein expression levels of 3+ or 2+, combined with FISH to confirm gene amplification. In this case, HER2 expression was not assessed through immunohistochemistry; instead, NGS revealed a mutation in exon 17 of the ERBB2 gene, where arginine was substituted by glutamine at position 678. This mutation indicated an alteration in the HER2 gene rather than gene amplification or increased HER2 protein expression. The limited efficacy of anti-HER2 ADC therapy in this patient may be associated with the absence of ERBB2 gene amplification.

Research has suggested that ERBB2 gene amplification and MSI-H are mutually exclusive in gastric cancer (13). Data from The Cancer Genome Atlas indicate that the MSI-H gastric cancer subtype exhibits a high mutation rate but lacks amplification of genes such as ERBB2 (13). Similarly, findings from the Asian Cancer Research Group (ACRG) showed that none of the 61 MSI-H gastric cancer cases studied exhibited ERBB2 gene amplification. Although MSI-H gastric cancer lacks ERBB2 gene amplification, studies have identified frequent mutations in the ERBB2 gene within this subtype (13). For instance, in HER2-mutated non-small cell lung cancer, the HER2 antibody-drug conjugate trastuzumab deruxtecan (T-DXd) has demonstrated durable anti-tumor activity (14). Investigating the efficacy of T-DXd in HER2-mutated advanced gastric cancer could offer promising treatment options. This approach may provide new insights for the patient’s subsequent therapy.

HER2-negative patients lack targets that can be targeted for therapy and could benefit from PD-1 and CTLA-4 bispecific antibodies. Antivascular therapy can improve hypoxia in the tumor microenvironment and reduce the recruitment of immunosuppressive cells thereby reversing the immunosuppressive tumor microenvironment. For patients with HER2-negative gastric cancer, antivascular drugs combined with cardunculizumab may become a new option after ICI resistance.

Many studies have reported a high frequency of KRAS mutations in MSI-H/dMMR GC, with activation of the KRAS-dependent pathway playing a significant role in the tumorigenesis of MSI-H/dMMR GC (15). While KRAS mutation is generally associated with poor prognosis, MSI-H status appears to counteract this effect, serving as a favorable prognostic factor in the presence of KRAS mutations (16). The PI3K/PTEN/Akt pathway is also more frequently dysregulated in MSI-H GC compared to MSS GC. Mutations in genes such as PIK3CA, PIK3R1, and PTEN lead to activation of this signaling pathway, which is critical to the pathogenesis of MSI-H tumors (17). Importantly, MSI-H/dMMR GCs harboring mutations in the PI3K pathway are reported to be responsive to PI3K pathway inhibitors (18). Given this patient’s KRAS and PIK3CA mutations, therapies targeting KRAS and PI3K pathway inhibitors could represent potential therapeutic options following resistance to existing treatments.

### 3.3 Immune checkpoint inhibitors and mechanisms of drug resistance

In this case, the metastatic lesions were significantly reduced following second-line treatment with chemotherapy combined with an ICI, and the efficacy was sustained for an extended period. The KEYNOTE-059, KEYNOTE-061, and KEYNOTE-062 studies investigated the efficacy of pembrolizumab in gastric cancer as third-line, second-line, and first-line treatments, respectively (19). These studies found that, in the MSI-H population, the pembrolizumab-treated group demonstrated higher ORR and OS compared to the chemotherapy group. Similarly, the 3-year follow-up results of the CheckMate-649 study revealed that in untreated MSI-H patients, the median OS was 38.7 months with nivolumab combined with chemotherapy, compared to 12.3 months with chemotherapy alone (HR=0.34) (20). These findings indicate that advanced MSI-H gastric cancer significantly benefits from ICI therapy due to its high mutation burden and unique immune microenvironment. However, the patient’s disease progression after 19 months of ICI therapy suggests the emergence of resistance to the treatment.

Most gastric cancer patients undergoing ICI therapy develop either primary or secondary resistance, with dMMR/MSI-H tumors being particularly prone to resistance due to their high immunogenicity (21, 22). Resistance to ICIs in dMMR/MSI-H tumors is influenced by tumor-intrinsic mechanisms, the tumor microenvironment, and host factors (23, 24). Tumor-intrinsic mechanisms include defective antigen-presenting cell function, depletion of neoantigens, and loss of genes associated with the interferon signaling pathway. Within the tumor microenvironment, factors such as reduced cytotoxic T-cell activity, an abnormal extracellular matrix, and suppression of T-cell function contribute to resistance in MSI-H gastric cancer. Host factors, including gut microbiota and dietary habits, also play a critical role in the development of resistance. Additionally, Wang et al. demonstrated a high mutation rate of genes in the PI3K-AKT-mTOR pathway in dMMR/MSI-H tumors (18). This mutation rate was negatively correlated with immune cell infiltration density, highlighting another potential mechanism underlying resistance to ICIs.

### 3.4 PD-1 and CTLA-4 bispecific antibodies for advanced gastric cancer

Cadonilimab, a bispecific antibody targeting both PD-1 and CTLA-4, represents a promising therapeutic option for advanced gastric cancer. CTLA-4, expressed exclusively on T-cells, acts as a negative regulator of T-cell activation by competitively binding to the CD28 co-stimulatory molecules CD80/CD86 on antigen-presenting cells (25, 26). While the PD-1/PD-L1 pathway suppresses anti-tumor T-cell responses at later stages, dual inhibition of these pathways may synergistically enhance anti-tumor immunity by blocking complementary mechanisms. In this case, cadonilimab was administered as a subsequent therapy after the patient developed resistance to PD-1 monoclonal antibody treatment. Although the primary tumor, liver metastases, and lymph nodes exhibited slight enlargement, they remained within a stable range. Following the addition of anti-vascular therapy, the patient achieved improved outcomes.

Cadonilimab has demonstrated remarkable efficacy in the first-line treatment of advanced gastric cancer. In the GEJC clinical study, the combination of cadonilimab and chemotherapy as first-line therapy for advanced gastric adenocarcinoma yielded a median OS of 17.41 months, a median PFS of 9.2 months, a 12-month OS rate of 61.4%, an ORR of 68.2%, and a DCR of 92% (3). In comparison, PD-1 monoclonal antibody combined with chemotherapy for first-line treatment in this patient population typically achieves a median OS of 10 to 12 months. However, the efficacy of cadonilimab in the rescue treatment of advanced MSI-H gastric cancer is not yet well established. A clinical trial (NCT04547101) is currently investigating cadonilimab in the second-line and beyond treatment of MSI-H/dMMR advanced tumors, and the results are eagerly awaited.

The patient developed a liver abscess during cadonilimab combined with anti-vascular therapy. A CT scan revealed a liver abscess measuring 108 mm × 96 mm, accompanied by fever and epigastric discomfort. Consequently, the patient underwent percutaneous tube drainage and antibiotic treatment. Following this intervention, the liver abscess significantly reduced in size, and the patient’s symptoms improved significantly, leading to a good recovery. Several factors may have contributed to the development of the liver abscess during treatment. Immunotherapy can potentially weaken the body’s bacterial defense mechanisms, increasing susceptibility to bacterial invasion and abscess formation in the liver. Additionally, local inflammation caused by tumor lysis syndrome may create an environment conducive to bacterial or microbial invasion, resulting in abscess formation. Tumor lysis syndrome may also increase vascular permeability, facilitating bacterial entry into liver tissue (27). Given the long-term and sustained immune memory effect of immunotherapy, cadonilimab was suspended for a total of four weeks during the management of the liver abscess, including drainage and antibiotic administration. A follow-up CT performed three months later showed that the metastatic and primary lesions remained stable compared to the previous imaging.

### 3.5 Antivascular treatment

Vascular endothelial growth factor (VEGF) and its receptor (VEGFR) are key in regulating tumor angiogenesis, promoting the proliferation and migration of vascular endothelial cells. Antivascular agents target VEGFR, effectively inhibiting tumor neovascularization and reducing tumor microvessel density. A study by Li et al. demonstrated that among patients with advanced gastric cancer who had undergone second-line or higher standard chemotherapy, those in the apatinib group had significantly improved OS and PFS compared to the placebo group, with minimal toxicity. As a result, apatinib is approved in China for third-line and later treatment of advanced gastric cancer.

In this case, the patient’s progression-free survival exceeded 20 months following salvage therapy with cadonilimab combined with apatinib. Numerous clinical studies have indicated that antivascular therapy can enhance the effectiveness of immunotherapy (28). At present, many clinical trials of anti-vascular therapy combined with immune checkpoint inhibitors in the treatment of advanced gastric cancer are under way (**Table 2**). Apatinib inhibits tumor angiogenesis, promotes T-cell infiltration into the tumor, and improves the suppressive microenvironment typical of MSI-H tumors, enabling greater infiltration of T cells into tumor tissues. When combined with a PD-1/CTLA-4 bispecific antibody, apatinib can further enhance the anti-tumor activity of these infiltrating T cells and increase tumor cell sensitivity to immunotherapy. Additionally, vascular normalization facilitated by antivascular therapy can improve immune cell permeability, further amplifying the impact of immunotherapy (4, 5).

**Table 2:** Clinical trials of anti-angiogenic drugs combined with immune checkpoint inhibitors in advanced gastric cancer.

Study	Treatment	Phase	Endpoint 1°	Population	Results
NCT03407976	Apatinib+Pembrolizumab	I/II	ORR, DLT	MSI-H/dMMR	NA
NCT04182724	Camrelizumab+Apatinib+Nab-paclitaxel	I/II	MTD/DLT, ORR	All	NA
NCT06592287	Adebrelimab+Apatinib+Lrinotecan liposome	I/II	PFS	All	NA
NCT04662710	Pembrolizumab+Lenvatinib+CAPOX/mFOLFOX6	III	PFS, OS	All	Part1:ORR 73%(11/15)DCR 93%(14/15)
NCT04792515	Camrelizumab+SOX/apatinib	II	PCR	All	NA
NCT04089657	Apatinib Mesylate+Sintilimab	II	DCR	All	NA
NCT04948125	Camrelizumab+Apatinib Mesylate	II	ORR	All	NA
NCT06238752	Apatinib+Tislelizumab+Chemotherapy	II	PFS	All	NA
NCT04195828	Camrelizumab+Apatinib Mesylate+Nab-paclitaxel+S-1	II	MPR	All	NA
NCT03609359	Lenvatinib+Pembrolizumab	II	ORR	All	NA
NCT05041153	Pembrolizumab +Lenvatinib	Early I	ORR	All	NA
NCT06383559	Lenvatinib+Sintilimab	II	ORR	All	NA
NCT04286711	Albumin-paclitaxel+Apatinib+Camrelizumab	I/II	DLT	All	NA
NCT04609176	Camrelizumab+Apatinb+SOX	II	ORR	All	NA

### 3.6 MSI-H gastric cancer salvage therapy

The low incidence of MSI-H gastric cancer has limited the availability of high-quality, large-scale, evidence-based studies for these patients, leaving no definitive recommendations for third- and later-line treatment options. The KEYNOTE-059 study assessed the efficacy and safety of pembrolizumab monotherapy in patients receiving third-line or higher treatment for advanced gastric cancer (29). The study reported an ORR of 47% in the MSI-H subgroup compared to 9% in the non-MSI-H gastric cancer group. Similarly, the CheckMate-032 trial evaluated nivolumab monotherapy and its combination with the anti-CTLA-4 antibody ipilimumab in patients with gastric cancer undergoing third-line or later treatments (30). In the MSI-H subgroup, the combination therapy group achieved an ORR and 18-month PFS rate of 50%, although with a significantly higher incidence of grade 3/4 adverse events. TAS-102 has shown promise in the third- and later-line treatment of advanced gastric cancer. The TAGS study demonstrated that TAS-102 significantly improved median OS compared to placebo in metastatic gastric cancer patients (5.7 months vs. 3.6 months) (31). Apatinib has also yielded favorable results for advanced gastric cancer in the third- and later-line setting. In a phase III trial, the apatinib-treated group had a significantly better OS compared to the placebo group (6.5 months vs. 4.7 months) (32). Clinical trials investigating the combination of antivascular therapy and immune checkpoint inhibitors for third- and later-line treatment of advanced MSI-H gastric cancer (NCT03407976, NCT04662710) are ongoing, and the data from these studies are highly anticipated.

## 4 Limitations

The patient did not undergo further immunohistochemistry testing to confirm HER2 protein expression status after the detection of the ERBB2 gene mutation. Additionally, due to financial constraints, the patient received cadonilimab at a dosage of 6 mg/kg every three weeks (q3w), while the standard dosage is 6 mg/kg every two weeks (q2w).

## 5 Conclusions

Immune checkpoint inhibitors (ICIs) have demonstrated significant therapeutic benefits for patients with microsatellite instability-high (MSI-H) gastric cancer. However, managing MSI-H tumors that develop resistance to ICIs remains a considerable challenge. In this case, the patient exhibited resistance to prolonged PD-1 monotherapy but subsequently achieved notable efficacy with a combination of PD-1/CTLA-4 dual-antibody therapy and apatinib. This outcome suggests that combining anti-vascular therapy with PD-1/CTLA-4 dual-antibody therapy may serve as a viable treatment option for MSI-H tumors resistant to ICIs. Further exploration into the application of this combined approach in MSI-H tumors is warranted.

## Data Availability

The original contributions presented in the study are included in the article/supplementary material. Further inquiries can be directed to the corresponding author.
